# A DFT Study on the Electronic Structures and Conducting Properties of Rubrene and its Derivatives in Organic Field-Effect Transistors

**DOI:** 10.1038/s41598-017-00410-6

**Published:** 2017-03-23

**Authors:** Huipeng Ma, Na Liu, Jin-Dou Huang

**Affiliations:** 1 College of Medical Laboratory Science, Dalian Medical University, Dalian, 116044 China; 2 Key Laboratory of New Energy and Rare Earth Resource Utilization of State Ethnic Affairs Commission, School of Physics and Materials Engineering, Dalian Nationalities University, Dalian, 116600 China; 3 State Key Laboratory of Molecular Reaction Dynamics, Dalian Institute of Chemical Physics, Chinese Academy of Sciences, Dalian, 116023 China

## Abstract

We systematically studied the electronic structures and conducting properties of rubrene and its derivatives reported recently, and disscussed the influences of electron-withdrawing groups and chemical oxidation on the reorganization energies, crystal packing, electronic couplings, and charge injection barrier of rubrene. Hirshfeld surface analysis and quantum-chemical calculations revealed that the introduction of CF_3_ groups into rubrene decreases the H···H repulsive interaction and increases intermolecular F···H/H···F attractive interactions, which resulted in the tight packing arrangement and the increase of the electronic couplings, and finally cause the higer intrinsic hole-mobility in bis(trifluoromethyl)-dimethyl-rubrene crystal (μ_h_ = 19.2 cm^2^ V^−1^ s^−1^) than in rubrene crystal (μ_h_ = 15.8 cm^2^ V^−1^ s^−1^). In comparison, chemical oxidation reduces charge-carrier mobility of rubrene crystal by 2~4 orders of magnitude and increased the hole and electron injection barrier, which partly explains the rubrene-based field-effect transistor performance degrades upon exposure to air. Furthermore, we also discussed the influence of structural parameters of carbon nanotube (CNT) electrode on charge injection process, which suggests that the regulation of CNT diameters and increasing in thickness is an effective strategy to optimize CNT work functions and improve n-type OFET performances based on these organic materials.

## Introduction

**Figure 1:**
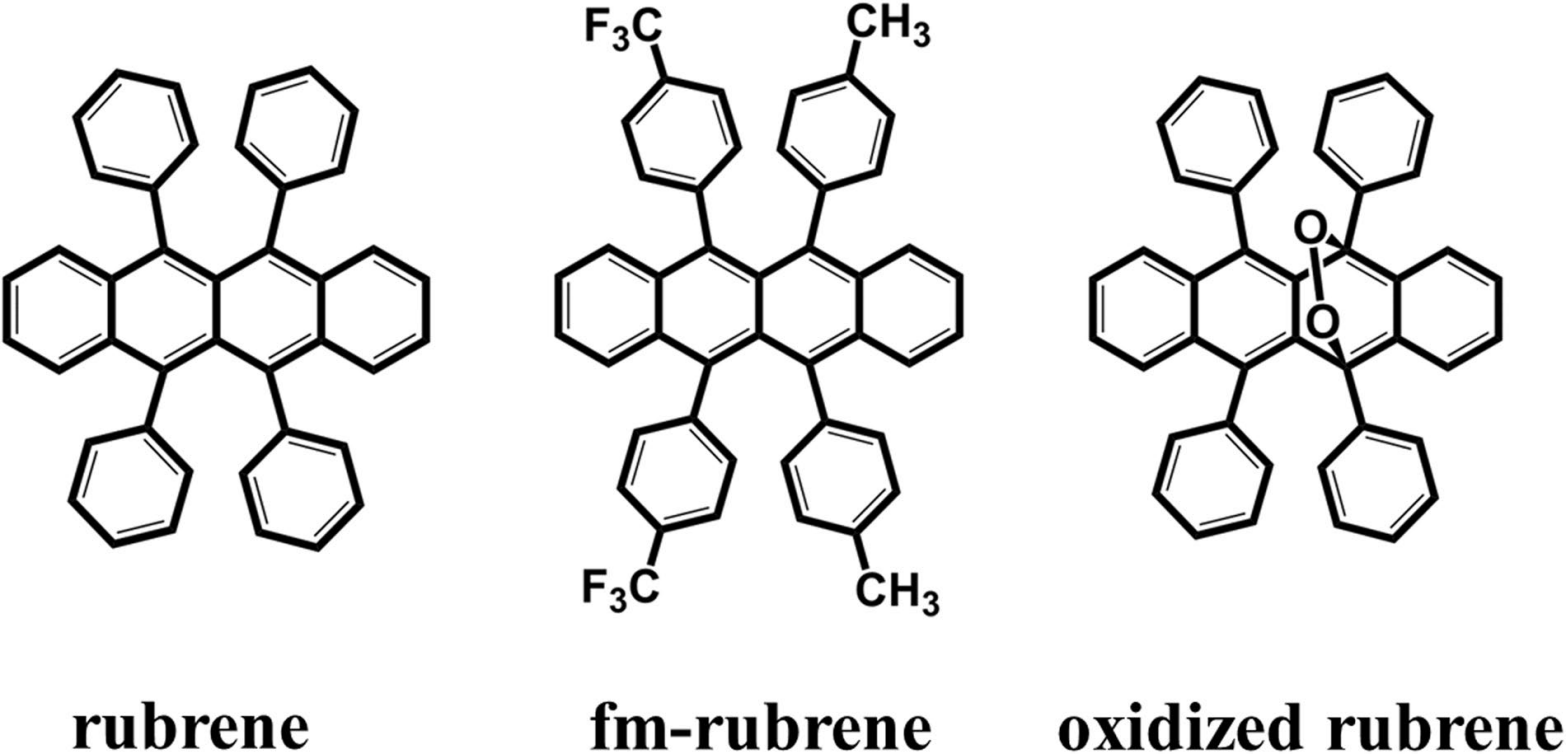
Molecular structures of rubrene, fm-rubrene, and oxidized rubrene.

As a prototypical organic semiconductor material, rubrene and its derivatives have attracted considerable attention due to their exemplary field-effect transistor properties^1–5^. Especially, room-temperature hole mobilities on the order of 15 cm^2^ V^−1^ s^−1^ could be achieved by fabricating field-effect transistors on the surface of rubrene single crystals^6^. These large mobilities have led to extensive rubrene-focused studies in an effort to explore more rubrene-based p-type and ambipolar organic materials. Recently, McGarry *et al*. synthesized a series of rubrene derivatives, and studied the effects of the molecular structure on solid-state packing and charge-transport properties^4^. Combined experimental and theoretical analysis, they concluded that fluoroalkyl substituents of rubrene should exhibit transport characteristics equivalent to, or in some cases improved on, those of the parent rubrene, as well as the potential for ambipolar behavior. Xie *et al*. further examined the ambipolar charge injection and transport properties of bottom contact single crystal field-effect transistors (SC-FETs) based on bis(trifluoromethyl)-dimethyl-rubrene (fm-rubrene, as shown in Fig. 1), by employing carbon nanotube (CNT) electrodes; and its benchmark ambipolar mobilities could be achieved, reaching 4.8 cm^2^ V^−1^ s^−1^ for hole transport and 4.2 cm^2^ V^−1^ s^−1^ for electron transport^5^.

Despite a series of rubrene-based organic semiconductors are synthesized, and structure−property relationship on rubrene derivatives have been investigated widely; the experimental design and development of rubrene-based materials are still in ‘try and error’ stage due to the limited understanding on the relationships between molecular structure and device performance. On the other hand, the determination of the intrinsic carrier mobility that reflects the conductive capacity of charge-carrier in ideal single-crystal structures is still a challenge to the present experimental methods. As one of the most important physical properties, the intrinsic anisotropic charge-carrier mobility provides reference for the optimization of field-effect mobility in organic electronic devices, and its value depends mainly on the molecular properties and molecular packing modes in theory, however, the experimental results of mobility are also affected by experimental conditions, such as measurement methods, microstructural characteristics of the dielectric layers, the semiconductor film-deposition temperature and so on^7, 8^, and thus the direct determination of the intrinsic carrier mobility though theoretical simulation method is an important and necessary task. So far, two common theoretical mechanisms, the band-like model and the hopping model, have been established to reveal the intrinsic charge transport processes in organic materials. These models make it possible to simulate and analyze the intrinsic carrier transport behavior in the crystals. In most semiconductor crystals formed by conjugated organic oligomers, the stacking and orientation of organic molecules are usually limited by weak van der Waals forces, which leads to the charge carriers localizing on a single molecule at the weak electronic coupling limit. Therefore, at ambient conditions, the hopping model is preferred for the prediction of carrier mobilities^9^. In the hopping model, several theoretical groups performed the analysis and prediction of the intrinsic transport behavior in the rubrene and pentacene crystals observed in organic field-effect transistor (OFET) experiments^10–13^. For examples, Wen *et al*. predicted the possible range of charge transfer rates in a rubrene single crystal and simulated the mobility anisotropy curve for rubrene; and the predicted anisotropic hole mobilities are in good agreement with the experimental results of Sundar *et al.* ^11^ However, the predicted range of mobility values implemented by anisotropic mobility analytical expressions combined with Marcus-Hush model^9^ was smaller than the current experimental measurements. Recently, Yin *et al*. developed the method of solving the master equation to simulate the anisotropy mobility of organic semiconducting materials, and the simulation method could give similar anisotropic character but more reasonable range of mobility for the systems with relatively large electronic couplings^13–16^. Herein, we adopt this new method to simulate the angle-resolved charge mobility of rubrene and its derivatives.

To deeply understand the effect of molecular structure on solid-state packing and resulting charge-carrier transport, we also systematically studied the electronic properties of rubrene and their derivatives (see Fig. 1), and discussed the influences of the electron-withdrawing group and chemical oxidation on the reorganization energies, molecular packing structures, electronic couplings, HOMO/LUMO distributions, electron affinity (EA) values and ionization energy (IP) values of rubrene. Besides, we also analyzed the influence of different carbon nanotube (CNT) structures on electron/hole injection barrier. Our theoretical investigations here could help to provide a fundamental understanding of substituent effect/chemical oxidation on rubrene and modification effect of CNT on metal electrode, thus enabling the rational design of high-performance OFETs with superior properties.

## Results and Discussion

### Reorganization Energies

As one of the key parameters influencing the intrinsic charge-transport rates, the reorganization energies evaluated from the four-point approach and from the normal-mode (NM) analysis are collected in Table 1. It can be seen that the reorganization energies calculated with NM analysis method are slightly larger than the ones evaluated from the four-point approach, which might originate from a clear deviation of the lattice vibration from the harmonic oscillator model. Comparison of these reorganization energies associated with intermolecular electron-transfer (λ_e_) and hole-transfer (λ_h_) shows that the introduction of CF_3_ groups and partial chemical oxidation of the rubrene push the λ_h_ and λ_e_ values to be larger than those of the unsubstituted rubrene. As show in Table 1, the λ_h_ value of fm-reburene and oxidized rubrene is 0.175 eV and 0.299 eV, respectively, which is about 0.07 eV and 0.20 eV larger than the one of rubrene molecule; the λ_e_ value of fm-rubrene and oxidized rubrene is 0.251 eV and 0.418 eV, respectively, and both are much larger than that of unsubstituted ruburene molecule (0.170 eV).

**Table 1:** DFT-B3LYP/6-311 G** calculated total reorganization energies of rubrene, fm-rubrene, and oxidized rubrene by the adiabatic potential surface (APS) approach and by normal mode analysis (NMA) method.

Molecular Crystals	λ_h_ (eV)		λ_e_ (eV)	
	NMA	APS	NMA	APS
rubrene	0.152	0.103	0.205	0.170
fm-rubrene	0.189	0.175	0.299	0.251
oxidized-rubrene	0.331	0.299	—	0.418

**Figure 2:**
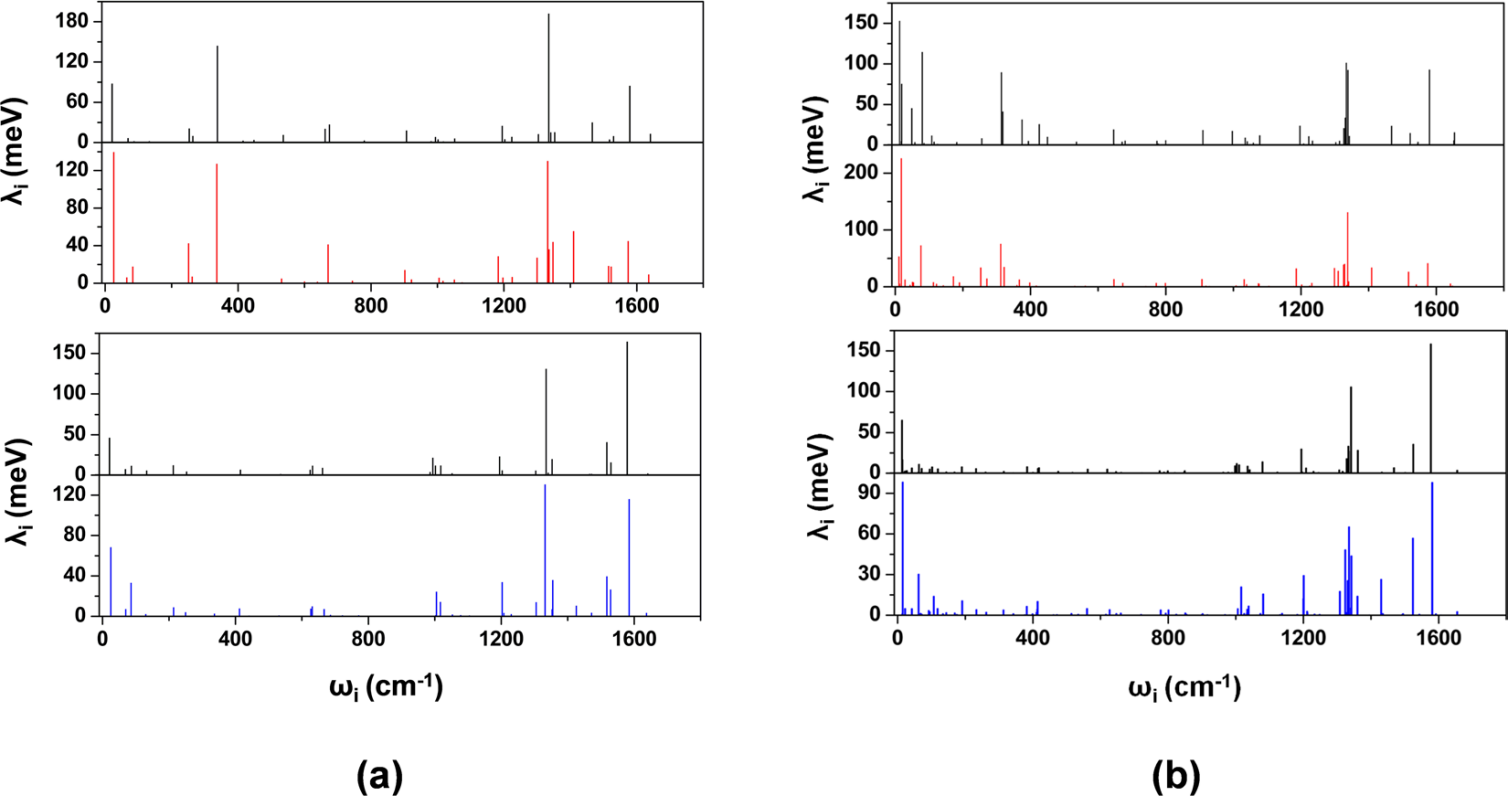
(**a**) Frequency dependence of reorganization energies for neutral and anionic rubrene upon electron-transfer process (top) and for neutral and cationic rubrene upon hole-transfer process (below); (**b**) Frequency dependence of reorganization energies for neutral and anionic fm-rubrene upon electron-transfer process (top) and for neutral and cationic fm-rubrene upon hole-transfer process (below); the black lines represent neutral compounds, the red lines represent anionic compounds, and the blue lines represent cationic compounds.

For sake of comprehensive analysis of the contributions of different vibration modes to the reorganization energy, we display the frequency dependence of mode specific reorganization energy in Fig. 2. Decomposition of the reorganization energy of rubrene into individual contributions from the relevant vibrational modes, as shown in Fig. 2a, indicates that both low-frequency and high-frequency modes contribute much to the electron-transfer reorganization energy: 46% of the total relaxation energy originates from vibrational modes at about 1200 cm^−1^ or higher, and 37% of the relaxation energy comes from low-frequency modes below 400 cm^−1^. In contrast, the contributions to the hole-transfer reorganization energy mainly come from high-frequency modes above 1200 cm^−1^ (see Fig. 2a). The calculation results show that 64% of the total relaxation involves vibrational modes about 1200 cm^−1^ or higher, and less than 16% comes from modes below 400 cm^−1^. Further analysis indicates that the low-frequency vibration modes contributing most to λ_h_ and λ_e_ values, such as 21 cm^−1^, 338 cm^−1^ in neutral molecule and 26 cm^−1^, 336 cm^−1^ in charged molecule, corresponds torsion vibration between four phenyl rings and naphthacene core; and the high-frequency vibration modes contributing most to λ_h_ and λ_e_ values, such as 1335 cm^−1^, 1579 cm^−1^ in neutral molecule and 1332 cm^−1^, 1574/1585 cm^−1^ in charged molecule, corresponds stretching vibration of chemical bonds in the naphthacene core. Thus, we can conclude that the bending vibration induced by variation of bond angles contributes more to the electron-transfer reorganization energy than the hole-transfer reorganization energy, as is similar with diphenyl-naphtho[2,3-b:6,7-b′]dithiophenematerial studied before^17^.

Figure 2b show the frequency dependence of reorganization energies for neutral and anionic fm-rubrene. It can be seen that the low-frequency (<400 cm^−1^) and high-frequency (>1200 cm^−1^) modes contribute most to the λ_e_ value, which is very similar with rubrene molecule. Our calculation results also show that the low-frequency modes contribute about 0.126 eV to the λ_e_ value of fm-rubrene, in comparison, the low-frequency modes of unsubstitued rubrene contribute about 0.063 eV to its λ_e_ value, which indicates that the introduction of CF3 groups significantly increase the contribution of the low-frequency modes to λ_e_ values. Further analysis shows that the low-frequency modes with the larger contributions to the λ_e_ value come from the vibration modes at 13 cm^−1^ in a neutral state and at 18 cm^−1^ in an anion state, both of which corresponds the bending vibration between CH_3_/CF_3_ substituted phenyl rings and naphthacene core. For hole-transfer process, our analysis results suggest that the introduction of CH_3_/CF_3_ groups increases λ_h_ value also by varying the contribution low-frequency modes. The low-frequency modes contribute about 0.046 eV to the total λ_h_ value of fm-rubrene, in contrast, the low-frequency modes contribute about 0.026 eV to the total λ_h_ value of rubrene; for the high-frequency modes, the vibration modes corresponding stretching vibration of C-F bonds in the molecule have few contribution to the total λ_h_ value. Therefore, for both λ_e_ and λ_h_, the contribution induced by electron-accepting affects of CF_3_ groups is larger than the direct contribution of C-F stretching vibrations.

Partial chemical oxidation is considered a more effective way to alter the parent compounds from being electron-donating to electron-accepting, as is observed in oligothienoacenes^18^; however, it also pushed the λ_h_ and λ_e_ values to be much larger than ones of the parent compound. Here, our calculation results show that the vibration modes corresponding torsion vibration and stretching vibrations of oxidative naphthacene core increase obviously (see Supporting Information Figure S1), and thus the total λ_e_ value and λ_h_ value of oxidized rubrene are much larger than the ones of rubrene. The observations are mainly related to the fact that the chemical oxidation of naphthacene core breaks the aromaticity of the oligomers and reduces the π-electron delocalization range, and also adds extra rotation degrees of freedom for geometric relaxation. Moreover, we also find that chemical oxidation of rubrene also leads to the increased hole and electron injection barrier, which is quite different with the observations in the oligothienoacenes. The relevant issues will be discussed in part *Ionization Potentials*, *Electronic Affinity*, *and Work Functions of Carbon Nanotubes*.

### Crystal structures and Electronic Couplings

The crystal structures of fm-rubrene and rubrene exhibit typical herringbone packing, and three types of intermolecular packing modes as T, P, and L can be defined in this case, as previous studied molecular systems^16–19^. The T and P dimers are in the same molecular stacking layer (shown in Figure S2), and head-to-tail stacking (L dimers) is out of the molecular stacking layer of T and P dimers. Considering the fact that the charge-transport between the layers was less efficient owing to the weak electronic couplings, and thus we mainly focused on the charge-transport within the basal-stacked organic layers. The calculated electronic couplings for hole- and electron-transfer (denoted as V_h_ and V_e_, separately), and the mass-centered distances r in T- and P-type dimers, are summarized in Table 2. Comparison of these effective electronic couplings and r values for the fm-rubrene and rubrene indicates that i) the V_h_ and V_e_ values in the fm-rubrene crystal are larger than the ones in rubrene crystal, especially for the P-type dimers; and ii) although the introduction of CF3/CH3 groups increase the volume of rubrene molecule to some extent, the r values in the fm-rubrene crystal are slightly shorter than the ones in rubrene crystal, which indicates that the addition of CF3/CH3 groups is favorable for forming a tight packing arrangement in the same layers.

**Table 2:** Calculated electronic coupling terms V_h_ (for hole-transfer) and V_e_ (for electron-transfer) for the different hopping pathways in rubrene, fm-rubrene and oxidized rubrene crystals; r is the intermolecular center-to-center distance.

Molecular crystals	Dimer types	r (Å)	V_h_ (meV)	V_e_ (meV)
rubrene	P1 = P2	7.174	89.3	58.8
	T1 = T2 = T3 = T4	8.021	16.0	8.3
fm-rubrene	P1 = P2	7.158	114.0	71.3
	T1 = T2 = T3 = T4	7.885	16.7	8.9
oxidized rubrene	1	7.078	0.3	19.7
	2	13.104	0.07	0.03
	3	11.092	0.4	11.0
	4	9.603	7.4	13.9
	5	15.939	0.0008	0.03
	6	13.212	0.1	5.5
	7	14.430	0.5	1.1
	8	11.259	0.4	1.1
	9	15.134	0.01	0.03

**Figure 3:**
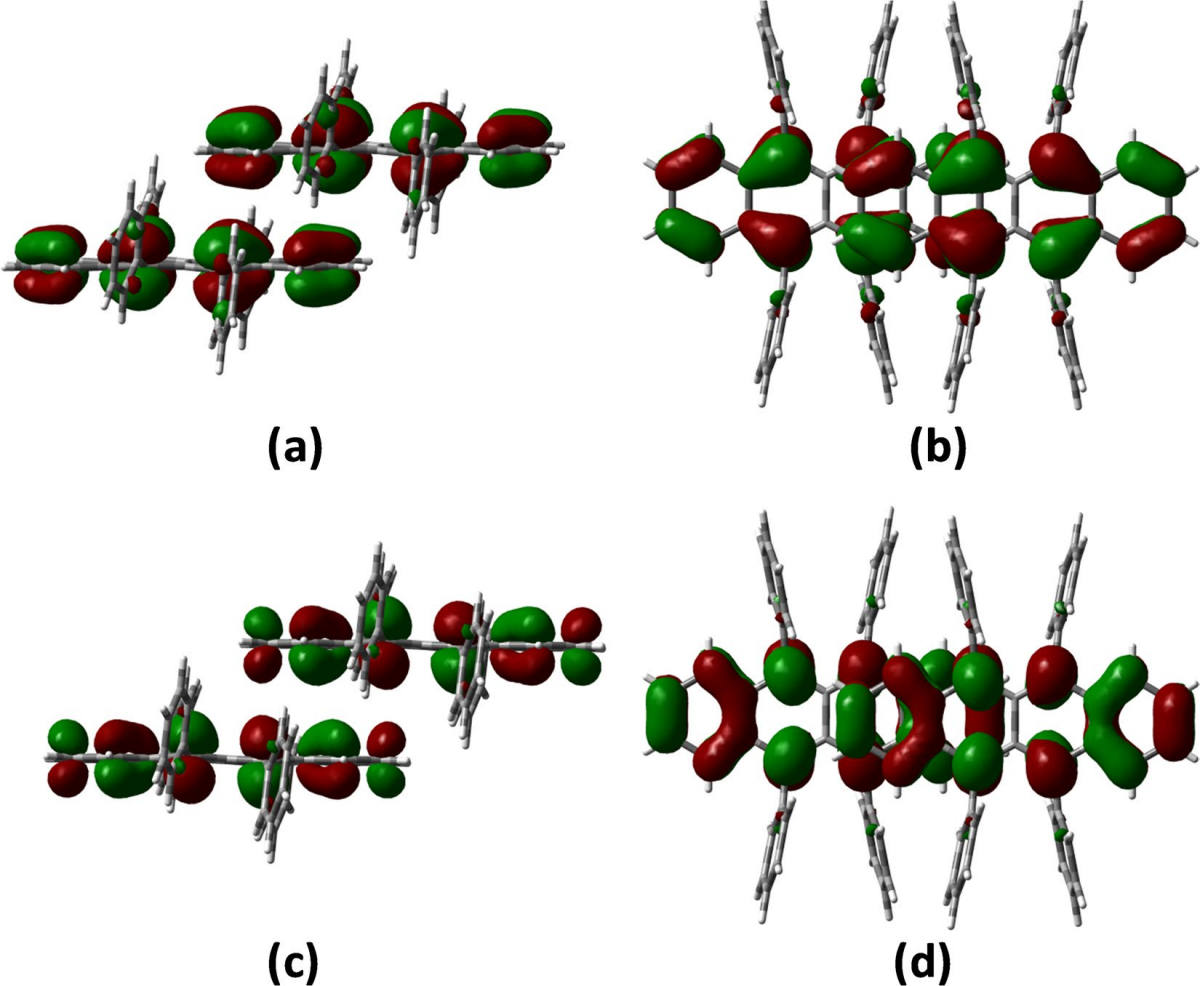
HOMOs (0.02 au) for P dimers of rubrene in side view (**a**), and in top view (**b**); and LUMOs (0.02 au) for P dimers of rubrene in side view (**c**), and in top view (**d**).

**Figure 4:**
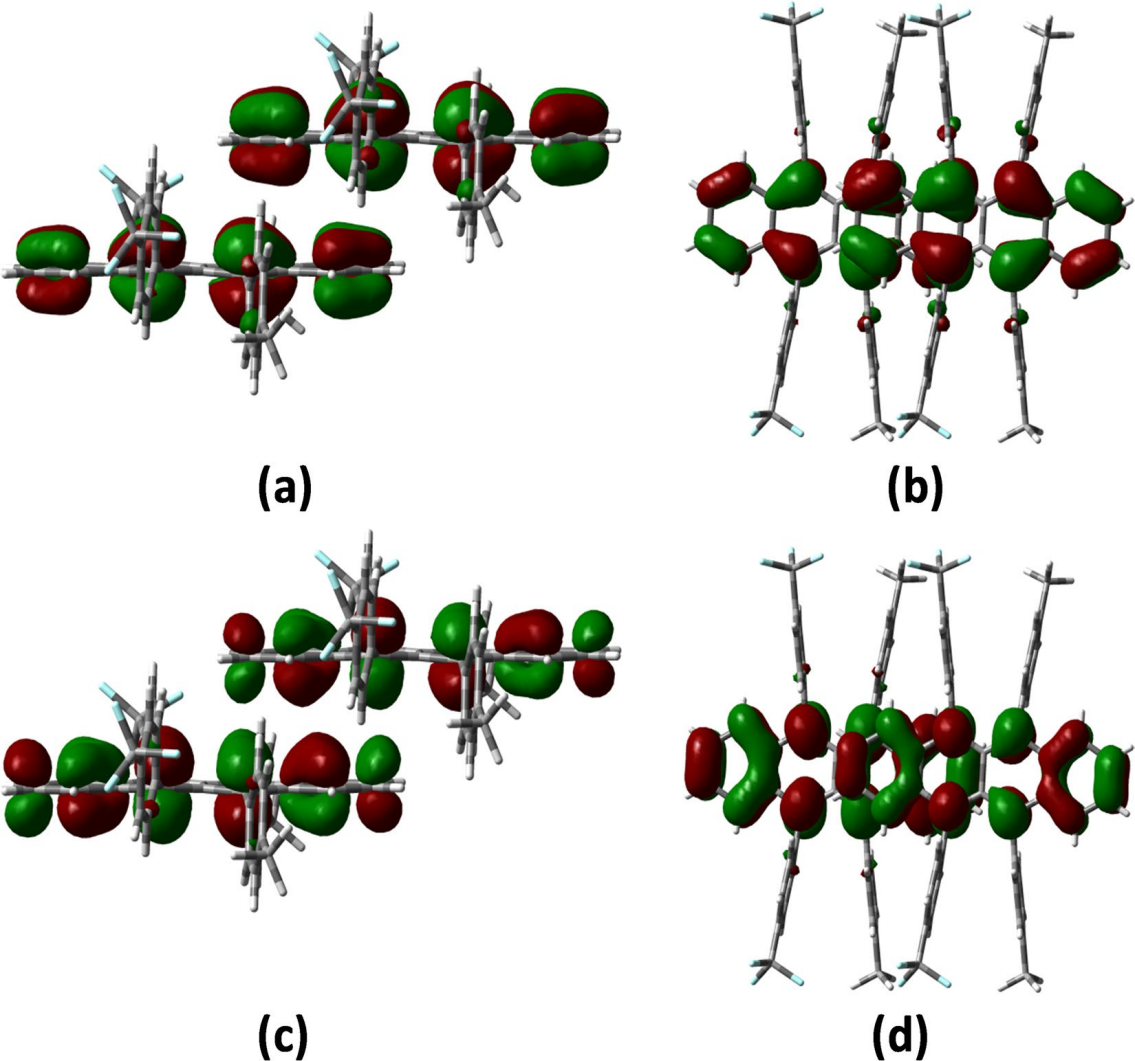
HOMOs (0.02 au) for P dimers of fm-rubrene in side view (**a**), and in top view (**b**); and LUMOs (0.02 au) for P dimers of fm-rubrene in side view (**c**), and in top view (**d**).

Figures 3 and 4, S3, and S4 shows the shapes of the HOMOs and LUMOs for rubrene and fm-rubrene, as well as packing structures of P and T dimers. We can see that the introduction of CF_3_ and CH_3_ has little influence on the frontier molecular orbital charge distributions, and for both rubrene and fm-rubrene the HOMO and LUMO mainly localized on the naphthacene core, which suggests the electronic couplings were determined by the relative positions of the naphthacene core part. As shown in Figs 3 and 4, there exists a relative displacement of about 2.5 benzene rings along the long molecular axis and nearly no displacement along the short molecular axis in the P dimer of rubrene and fm-rubrene, and their similar face-to-face packing structures result in the approximate effective coupling projected areas^20^ in rubrene and fm-rubrene, and thus the larger electronic couplings in P dimers of fm-rubrene are mainly related to the relatively smaller separate distance between two fm-rubrene molecules. In the T dimer, the edge-to-face packing motifs lead to a small effective coupling projected area (see Figures S3 and S4), which rationalizes much smaller electronic couplings in T dimer than in P dimer.

Another important factor affecting the electronic couplings is the shapes of the frontier molecular orbitals. As show in Figs 3 and 4, the HOMOs of rubrene and fm-rubrene are mainly located on C-C bonds that aligned predominantly along the long molecular axis, and shows approximate uniform spatial distribution. These distribution characteristics suggest that there exits obvious bonding or antibonding overlaps between the π-atomic orbitals in P dimer, although it is partly reduced by relative displacement along the long molecular axis. In comparison, the LUMO localized on naphthacene core show an uneven distribution. As shown in Figs 3 and 4, the LUMOs located on the bilateral benzene rings show quite different distribution characters with these located on the central benzene rings, for example, the central C-C bond that parallels to the short molecular axis shows non-bonding character, while the other C-C bonds show typical bonding character. These distribution characters dramatically decrease the global overlap between the LUMO levels in the P dimer, which well explains the larger V_h_ than V_e_ values in P dimer.

**Figure 5:**
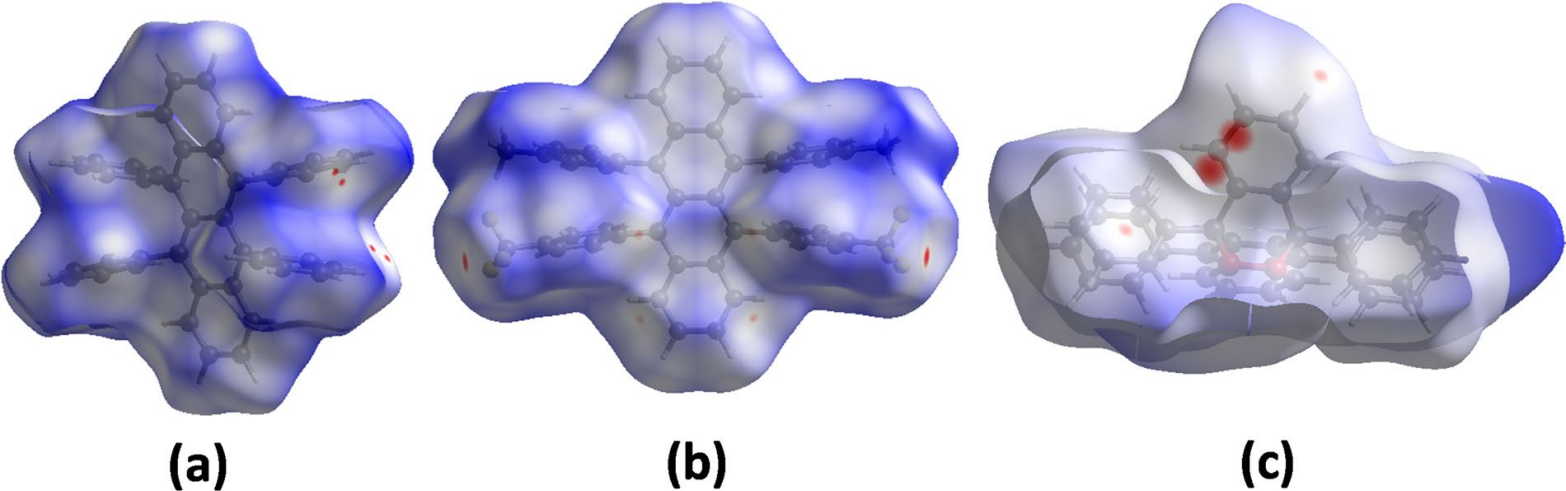
Hirshfeld surfaces mapped with normalized contact distance (d_norm_) for rubrene (**a**), fm-rubrene (**b**), and oxidized rubrene (**c**).

In order to elucidate the influence of CF_3_/CH_3_ groups on intermolecular interactions and molecular packing structures, we performed Hirshfeld surfaces on the present rubrene and fm-rubrene crystals^21, 22^. The three-dimensional (3D) Hirshfeld surfaces of these two molecules are shown in Fig. 5; they clearly show the influences of CF_3_/CH_3_ groups on the intermolecular interactions. For the rubrene molecule, as shown in Fig. 5a, the small red cycles on the two ends of the 3D Hirshfeld surfaces represent H···H interactions between two-dimensional (2D) molecular layers (MLs), which indicates that the shortest separation distance is below the sum of the van der Waals (vdW) radii of two hydrogen atoms. In the crystal of rubrene, the H···H interactions within ML and between MLs have the most significant contribution to the total Hirshfeld surfaces of rubrene, comprised of 74.3% (see Figure S5), and these H···H repulsive interactions increase the mass-centered distances between the neighbouring monimers to some extent. For fm-rubrene molecule, the large red cycles visible on the 3D Hirshfeld surfaces near CF_3_ groups (see Fig. 5b) are corresponding to the F···F interaction between 2D molecular layers, comprises 4.5% of the total Hirshfeld surfaces; the small red cycles on the terminal of the naphthacene core represent the H···H interactions in the same ML. In the crystal of fm-rubrene, the contribution of the H···H interactions to the total Hirshfeld surface obviously decrease, comprised of 50.3%, and thus the affect of H···H repulsive interaction on the mass-centered distances is much weakened. It is also noteworthy that the F···H/H···F interactions in fm-rubrene crystal also have a relatively significant contribution to the total Hirshfeld surfaces, comprised of 23.4% (see Figure S5), and these interactions include intramolecular and intermolecular F···H/H···F interactions between MLs and in the same MLs. Of these vdW interactions, the intermolecular F···H/H···F interactions have a significant contribution to the tight packing arrangement of fm-rubrene molecules and large effective electronic couplings between the neighboring molecules. To evaluate the contributions of the intermolecular F···H/H···F interactions to the total binding energies, we have computed the reactions shown in Supporting Information Figure S6. For rubrene and fm-rubrene, the strength of the H···π interaction is almost consistent, and the difference is mainly observed in the F···H interaction. Therefore, the large differences observed in the formation energies of the dimers (204 kcal/mole for P dimer, and 206.4 kcal/mole for T dimer) mainly reflect contribution of the F···H interaction.

The Hirshfeld surfaces of oxidized rubrene are illustrated in Fig. 5c. The large circular depressions (deep red) visible on the Hirshfeld surfaces are indicative of intermolecular π···π (C···C) interactions, comprises 2.3% of the total Hirshfeld surfaces, and other visible bright red spots are due to intermolecular C···H contacts (comprised of 26.7%). Similar with rubrene and fm-rubrene, the H···H interactions, which can be viewed in Hirshfeld surface plots by the white area (used for contacts around the vdW separation) in Fig. 5c, have the most significant contribution to the total Hirshfeld surfaces, comprised of 65%. Apart from those above, the presence of C-H···O interactions are observed, which comprise 6.0% of the total Hirshfeld surface. Further comparison of oxidized rubrene and rubrene suggests that the partial oxidation not only induce intermolecular C-H···O interactions in the crystal, but also increase the relative contributions of the C···H interactions to the Hirshfeld surfaces due to the flexible carbon skeleton and suitable orientation degrees of freedom, and these variations in the intermolecular interactions leads to diverse crystal packing arrangements.

### Anisotropic Mobilities

**Figure 6:**
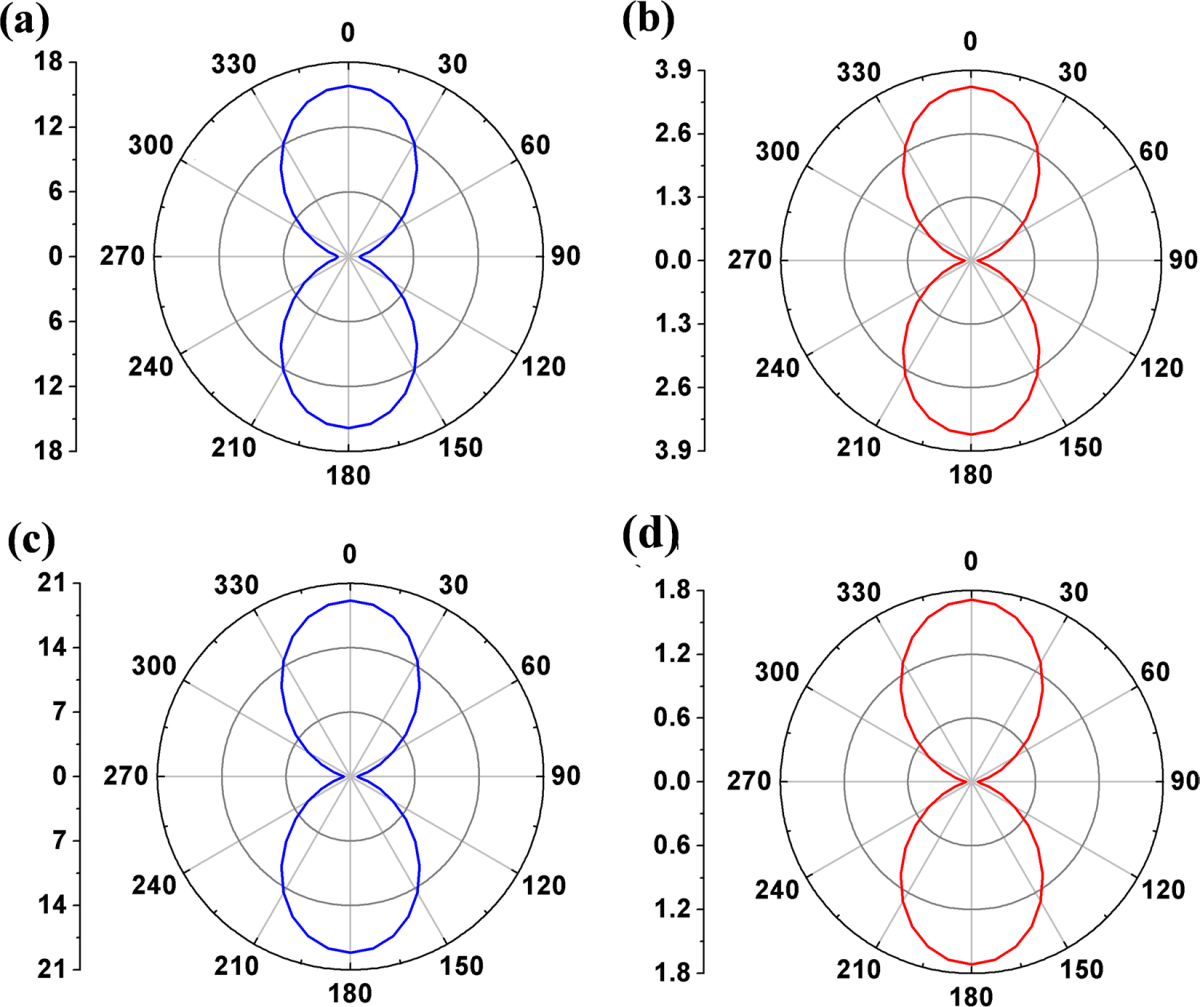
Calculated angle-resolved anisotropic hole mobilities (**a**) and electron mobilities of rubrene (**b**); and calculated angle-resolved anisotropic hole mobilities (**c**) and electron mobilities of fm-rubrene (**d**); here, 0° corresponds crystallographic axis b, and 90° corresponds crystallographic axis c.

The anisotropic hole-transfer and electron-transfer mobilities in the single crystals of rubrene and fm-rubrene are shown in Fig. 6. It can be seen that their similar crystals structures result in the same angle dependence of mobility: both rubrene and fm-rubrene shows remarkable anisotropic behaviour and the highest mobility value appears when the value of Φ is near 0°/180° (crystallographic axis b direction), which is consistent with their larger hole- and electron-transfer integrals in P dimers than other dimers. The ranges of mobility values of rubrene and fm-rubrene estimated in the same layer are summarized in Table 3. We can see that the ranges of the hole/electron mobility in rubrene and fm-rubrene crystals agree well with the recent experimental measurements^5^, which verifies the rationality of our computation method and strategy. Comparison of the theoretical hole and electronic mobility values for rubrene and fm-rubrene indicates that the holes in fm-rubrene are intrinsically more mobile than the holes in rubrene; while for the electron, the mobility values in the crystal of fm-rubrene is obviously lower than that in the rubrene. Combined the experimental results reported recently, we could conclude that the current experimentally mobility of fm-rubrene is about 1/6th of its optimum value and thus further effort could likely improve the hole mobility by a factor of 6; in comparison, the electron-transfer mobility of fm-rubrene might have reached its optimum value as a type of n-type or ambipolar materials.

**Table 3:** Theoretical hole-diffusion mobilities (μ_h_) and electron-diffusion mobilities (μ_e_) of rubrene, fm-rubrene and oxidized rubrene at room temperature (T = 300 K), and some experimental charge-transfer mobility values.

	μ_h_ (theory)	μ_h_ (experimental)	μ_e_ (theory)	μ_e_ (experrimental)
rubrene	1.0–15.8	7.07 ± 1.77^5^	0.1–3. 6	
fm-rubrene	0.7–19.2	2.86 ± 1.11^5^	0.05–1.7	1.37 ± 0.84^5^
oxidized rubrene (ac plane)	0.001–0.09		1.0*10^−5^–1.6*10^−4^	
oxidized rubrene (bc plane)	0.09–0.12		(1.9–5.4)*10^−5^	

The complicated crystal structure of oxidized rubrene leads to two different conducting 2D layers: one is on the *ac* plane, and the other is on the *bc* plane. We separately simulated their angular resolution anisotropic mobility for both electron- and hole-transport. As shown in Figure S7a and b, it can be seen that both hole- and electron-transfer mobility values in the *ac* plane show remarkable anisotropic behavior: for the hole-transfer, the highest (0.09 cm^2^ s^−1^ V^−1^) and lowest mobility values (0.001 cm^2^ s^−1^ V^−1^) present at Φ = 155°/335° and Φ = 65°/235°, respectively; and for the electron-transfer process, the optimum charge-carrier transport direction is along Φ = 100°/280°, and the direction along Φ = 10°/190° shows the lowest mobility values. These anisotropic behaviours indicate that the selection of suitable conduction channel is very important for the OFET properties of oxidized rubrene. The predicted mobility anisotropy curves for hole-transfer and electron-transfer in *bc* plane are depicted in Figure S7c and d, and they shows similar anisotropic behaviour: both the maximum hole mobility value (0.12 cm^2^ s^−1^ V^−1^) and the maximum electron mobility value (0.00005 cm^2^ s^−1^ V^−1^) appear at Φ = 0°/180°, and the corresponding lowest hole and electron mobilities appear near Φ = 90°/270°, which suggests that the optimum values of electron- and hole-transfer mobility appear near dimer ***1*** direction due to the relatively larger electronic coupling. By comparison of hole/electron mobility values of oxidized rubrene with those of rubrene, it found that the charge-carrier mobility of oxidized rubrene is about 2 to 4 orders of magnitude lower than the ones of rubrene, which shows the detrimental effect of oxygen incorporation on transport properties of rubrene crystal.

### Ionization Potentials, Electronic Affinity, and Work Functions of Carbon Nanotubes

Besides the mobility, the charge injection efficiency is also an important factor that affects the performance of OFET device, especially for the ambipolar and n-channel OFETs^15, 23–25^. For OFETs, it demands that the electrode materials have the work functions suited for injection of holes/electrons into the HOMO/LUMO of semiconductor molecules, namely, the electron affinity/ionization potential of organic semiconductor is high/low enough to allow efficient injection of electrons/holes into empty LUMO/HOMO. Here, we calculated IPs, EAs, HOMOs, and LUMOs of rubrene, fm-rubrene, and oxidized rubrene, as shown in Table 4. In molecular orbital theory approaches, the HOMO energy is related to the IP by Koopmanns’ theorem and the LUMO energy has been used to estimate the electron affinity (−E_HOMO_ = IP and −E_LUMO_ = EA), however, the −E_HOMO_/−E_LUMO_ values is usually inconsistent with IP/EA values in the practical DFT calculations partly due to the unknown “exact” exchange-correlation functional. Previous calculations by Zhan *et al*. showed that the directly calculated vertical IPs are, on the whole, in good agreement with the corresponding experimental IPs, in contrast, the negatives of the HOMO energies, calculated with both the smaller and larger basis sets are all systematically smaller than the experimental and calculated IPs^26^. Thus, we select VIP values and EA values of the studied compounds and the work functions of different carbon nanotubes (CNTs) as the evaluation parameters to discuss the effects of molecular modification and CNT electrodes on the charge injection process.

**Table 4:** VIPs, AIPs, VEAs, AEAs, HOMO energy levels, and LUMO energy levels calculated at the B3LYP/6–311** level (eV).

Molecular Crystals	VIP	AIP	VEA	AEA	HOMO	LUMO
rubrene	6.11	6.05	1.37	1.31	−4.94	−2.34
oxidized-rubrene	7.15	7.01	0.60	0.38	−5.94	−1.38
fm-rubrene	6.28	6.20	1.70	1.55	−5.15	−2.63

For the bare Au electrode, the key for the efficient injection of charge-carrier is that the VIP values should be close to or smaller than the Au work function (5.0 eV), namely, HOMO levels align close to or higher than −5.0 eV; and the EA values should be close to or larger than the Au work function, and thus the LUMO levels align close to or lower than −5.0 eV. Considering the thermal and oxidative stability of electron-transport materials, the suitable EA values need to be at least 3.0 eV, but should not be much greater than 5.0 eV. Comparison of rubrene, fm-rubrene, and oxidized rubrene shows that i) the introduction of electron withdrawing group CF_3_ improve both VIP value and EA value of parent molecule, and thus the hole injection barrier increases and the electron injection barrier decrease, which is favourable for the transformation from p-type materials to ambipolar or n-type materials; ii) partial chemical oxidation of the rubrene improve VIP value and reduce EA value, and thus the injection barrier of both hole and electron increases, the injection efficiency of charge-carrier is greatly decreased, which well explains the susceptibility of organic materials to atmospheric oxidants^27, 28^.

**Figure 7:**
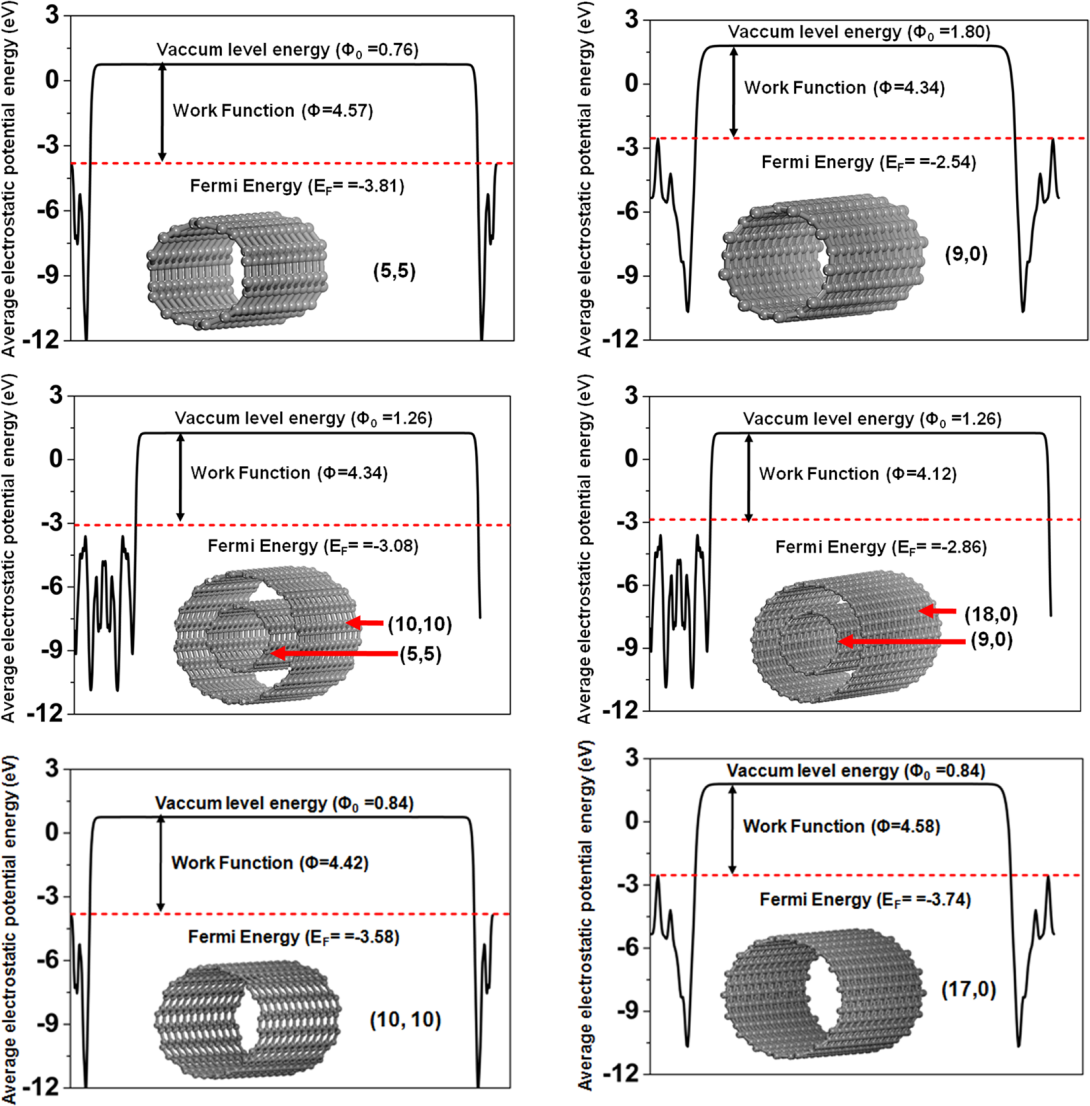
Calculated work functions of armchair-type single-walled CNTs (5, 5), (9, 0), zigzag-type single-walled CNTs (10, 10), (17, 0), and multi-walled CNTs (5, 5)/(10, 10), and (9, 0)/(17, 0).

As a surface modification material, CNTs have been regarded as great candidates for future nanoscale electronic and photonic devices and it has also been shown by experiments that work functions of CNTs are crucial for performance of CNT-based field effect transistors and diodes^29–31^. Herein, we selected armchair-type single-walled CNTs (5, 5), (9, 0), zigzag-type single-walled CNTs (10, 10), (17, 0), and multi-walled CNTs (5, 5)/(10, 10), and (9, 0)/(17, 0) as theoretical models, and simulated their work functions by density function theory calculations. The calculation results are collected in Fig. 7. We can seen that the work functions of all CNTs are lower than Au electrode, and could effectively reduce electron injection barrier, at same time, the stability of CNTs overcome shortcomings of low-workfunction metals, such as easy-oxidation, formation of reactive complexes with the organic semiconductor. Further analysis shows that the work functions of CNTs vary as a function of their dimeters and show different variation tendency for armchair-type and zigzag-type CNTs. The work functions of armchair CNTs slightly decrease with increasing tube diameters, while the work functions for zigzag-type CNTs slightly increase with increasing tube diameters. As shown in Fig. 7, the work function of CNT (5, 5) is 4.57 eV, about 0.15 eV higher than the work function of CNT (10, 10); for CNT (9, 0) the work function is 4.34 eV, which is about 0.24 eV lower than the one of CNT (17, 0). Most interestingly, the multi-welled CNT consists of two nested single-walled CNTs shows lower work functions than the ones of any single-walled CNTs. As shown in Fig. 7, the work function of multi-walled CNT (5, 5)/(10, 10) is about 4.34 eV, which is lower than the ones of single-walled CNT (5, 5) and single-walled CNT (10, 10); and the work function of multi-walled CNT (9, 0)/(17, 0) is about 4.12 eV, which is 0.22 eV and 0.46 eV lower than the ones of single-walled CNT (9, 0) and single-walled CNT (17, 0), respectively. These calculation results indicate that the control and regulation of CNT wall thickness is also an effective strategy to optimize OFET performances.

## Conclusions

In this manuscript, we simulated anisotropic charge-transfer mobilities of rubrene, fm-rubrene, and oxidized rubrene, and theoretically predicted the range of their mobility values, which provide reference for the performance optimization of OFET based on these materials. We systematically studied the influences of electron-withdrawing group CF_3_ and chemical oxidation on the reorganization energies, crystal packing, electronic couplings, and charge injection barrier of rubrene. It is found that the introduction of CF_3_ groups into rubrene increases the reorganization energy of rubrene molecule, but decreases the H···H repulsive interaction and increases intermolecular F···H/H···F attractive interactions, which resulted in the tight packing arrangement and the increase of the electronic couplings between neighbor monomers. As a result, the holes in fm-rubrene crystal are intrinsically more mobile than the holes in rubrene crystal. Moreover, the introduction of CF_3_ groups also induced an obvious increase in the vertical electronic affinities and the vertical ionization potentials, which is favourable for the transformation from p-type materials to ambipolar or n-type materials. The chemical oxidization of the ruberne molecule leads to significantly large reorganization energy and complex molecular packing structures; more importantly, it improved VIP value and declined EA value, which increase the injection barrier of both hole and electron, and thus greatly decreases the injection efficiency of charge-carrier, therefore, chemical oxidization of naphthacene core is harmful for the performance improvement of rubrene-based materials. Furthermore, we discuss and analyze the relationship between work functions and CNT wall thickness and diameter, which indicates the regulation of CNT diameters and increasing in thickness is an effective strategy to optimize CNT work functions and n-type OFET performances.

## Computational Methods

The reorganization energy λ associated with charge transport process in organic solid materials can be evaluated in two ways. The first is the normal-mode (NM) analysis method, which provides the partition of the total relaxation energy into the contributions from each vibrational mode:

1 $${\rm{\lambda }}={{\rm{\Sigma }}{\rm{\lambda }}}_{{\rm{i}}}={\rm{\Sigma }}({{\rm{\omega }}}_{{\rm{i}}}^{2}\ast {{\rm{\Delta }}{\rm{Q}}}_{{\rm{i}}}^{2})/2,$$

where ΔQ_i_ represents the displacement along normal mode Q_i_ between the equilibrium geometries of the neutral and charged molecules; ω_i_ is the corresponding frequency. The NM analysis is performed with the DUSHIN program^32^ combined with calculation results from Gaussian 03. The other method isthe adiabatic potential-energy surface method (the four-point approach), in which the λ can be expressed as follows:

2 $${\rm{\lambda }}={{\rm{\lambda }}}_{1}+{{\rm{\lambda }}}_{2}=({{\rm{E}}}_{\pm }^{\ast }-{{\rm{E}}}_{\pm })+({{\rm{E}}}^{\ast }-{\rm{E}}).$$

Here, E and E_±_ represent the energies of the neutral and cation/anion species in their lowest energy geometries, respectively; E^*^ and ${{\rm{E}}}_{\pm }^{\ast }$ are the energies of the neutral and cation/anion species with the geometries of the cation/anion and neutral species, respectively. From the adiabatic potential-energy surfaces of neutral/charged species, the vertical ionization potential (VIP), adiabatic ionization potential (AIP), vertical electronic affinity (VEA), and adiabatic eledctron affinity (AEA) can be calculated as:

3 $${\rm{VIP}}={{\rm{E}}}_{+}^{\ast }-{\rm{E}},$$

4 $${\rm{AIP}}={{\rm{E}}}_{+}-{\rm{E}},$$

5 $${\rm{VEA}}={\rm{E}}-{{\rm{E}}}_{-}^{\ast },$$

6 $${\rm{AEA}}={\rm{E}}-{{\rm{E}}}_{-}.$$

Full geometry optimizations of the monomer molecules and the reorganization energy calculations are carried out using the B3LYP functional in conjunction with the 6–311 G** basis set. These calculations are performed with the Gaussian 03 package^33^.

The intermolecular electronic coupling V_ij_, which describes the overlap of electronic wave functions between the donor and acceptor states, can be written as:

7 $${{\rm{V}}}_{{\rm{ij}}}=|({{\rm{J}}}_{{\rm{ij}}}-0.5\ast ({{\rm{e}}}_{{\rm{i}}}+{{\rm{e}}}_{{\rm{j}}})\ast {{\rm{S}}}_{{\rm{ij}}})/(1-{{\rm{S}}}_{{\rm{ij}}}^{2})|,$$

where S_ij_, J_ij_, and e_i(j)_ respectively represent the spatialoverlap, charge transfer integrals, and site energies. These physical quantities can be calculated as follows:

8 $${{\rm{e}}}_{{\rm{i}}({\rm{j}})}=\langle {{\rm{\Psi }}}_{{\rm{i}}({\rm{j}})}|{\rm{H}}|{{\rm{\Psi }}}_{{\rm{i}}({\rm{j}})}\rangle ,$$

9 $${{\rm{S}}}_{{\rm{ij}}}=\langle {{\rm{\Psi }}}_{{\rm{i}}}|{{\rm{\Psi }}}_{{\rm{j}}}\rangle ,$$

10 $${{\rm{J}}}_{{\rm{ij}}}=\langle {{\rm{\Psi }}}_{{\rm{i}}}|{\rm{H}}|{{\rm{\Psi }}}_{{\rm{j}}}\rangle .$$

Here, H is the system Kohn-Sham Hamiltonian of the dimer system, and Ψ_i(j)_ means the monomer HOMOs (for hole transport) or LUMOs (for electron transport) with Löwdin’s symmetric transformation which can be used as the orthogonal basis set for calculation. The calculations of all electronic couplings in different molecular dimers are performed with the PW91/TZ2P of density functional theory (DFT) implemented in the Amsterdam density functional (ADF) program^34^.

The anisotropic mobility is an import intrinsic property of the charge transport in organic semiconductors, which depends significantly on the specific surface of organic crystals. Herein, we simulated the angle-resolved charge mobility of rubrene and its derivatives by means of solving the master equation, whichhas been described in detail elsewhere^13, 14, 35–38^. The charge-transfer (CT) kinetics through the solid material with many possible residence sites can be described by the master equation.

11 $$\frac{{{\rm{dp}}}_{{\rm{i}}}}{{\rm{dt}}}=-\sum _{{\rm{j}}\ne {\rm{i}}}[{{\rm{k}}}_{{\rm{ij}}}{{\rm{p}}}_{{\rm{i}}}(1-{{\rm{p}}}_{{\rm{j}}})-{{\rm{k}}}_{{\rm{ji}}}{{\rm{p}}}_{{\rm{j}}}(1-{{\rm{p}}}_{{\rm{i}}})]$$

where k_ij_ is the CT rate constant from site i to site j in the crystal considering the correction of the electronic field, p_i_ is the charge occupied density on site i, and 1−p_i_ is the Coulomb penalty factor, which prevents two or more charges at the same time from occupying the same site. If the CT reaches to the so-called steady state, dp_i_/dt = 0, the p_i_ can be obtained by an efficient iterative procedure given a full set of CT constant k_ij_. When an external electronic field E is applied to the crystal, the charge will drift accordingly, and the charge mobility m can be determined from the velocity v as the linear response of the motion to the perturbation: ${\rm{\mu }}=\frac{{\rm{v}}}{|{\rm{E}}|}=\frac{{\sum }_{{\rm{ij}}}{{\rm{k}}}_{{\rm{ij}}}{{\rm{p}}}_{{\rm{i}}}(1-{{\rm{p}}}_{{\rm{j}}}){{\rm{R}}}_{{\rm{ji}}}\widehat{{\rm{E}}}}{{{\rm{p}}}_{{\rm{tol}}}|{\rm{E}}|}$ where $\widehat{{\rm{E}}}$ is the unit vector of applied electric field, R_ji_ is the vector from site i to site j, and p_tol_ is the total charge population in the investigated supercell. The calculation here is performed using the periodic boundary condition with a supercell of size 3 × 3 × 3, and the external electric field E is set to a relatively small value of ${\bf{1.0}}\ast {{\bf{10}}}^{{\boldsymbol{-}}{\bf{3}}}{\rm{V}}{\AA }^{{\boldsymbol{-}}{\bf{1}}}$.

## Electronic supplementary material

Supplementary Informations
