# Supplementary material for: A DFT Study on the Electronic Structures and Conducting Properties of Rubrene and its Derivatives in Organic Field-Effect Transistors
Source: Sci Rep. 2017 Mar 23;7:331. doi: 10.1038/s41598-017-00410-6 (PMC5428530; doi:10.1038/s41598-017-00410-6)
Supplement: Supplementary file 1 — Supplementary Informations [file 41598_2017_410_MOESM1_ESM.doc]

**Electronic Supplementary Information**

A DFT Study on the Electronic Structures and Conducting Properties of Rubrene and its Derivatives in Organic Field-Effect Transistors

Huipeng Ma^1*^, Na Liu,^1^ and Jin-Dou Huang^2,3*^

^1^ College of Medical Laboratory Science, Dalian Medical University, Dalian 116044, China.

^2^ Key Laboratory of New Energy and Rare Earth Resource Utilization of State Ethnic Affairs Commission, School of Physics and Materials Engineering, Dalian Nationalities University, Dalian, 116600, China.

^3^ State Key Laboratory of Molecular Reaction Dynamics, Dalian Institute of Chemical Physics, Chinese Academy of Sciences, Dalian 116023, China.

*Corresponding author. Tel: +86 41186110391. E-mail: hpma@dlmedu.edu.cn (Huipeng Ma)

* Corresponding author. Tel: +86 41187658872. E-mail: jindouhuang@dicp.ac.cn. (Jin-Dou Huang)


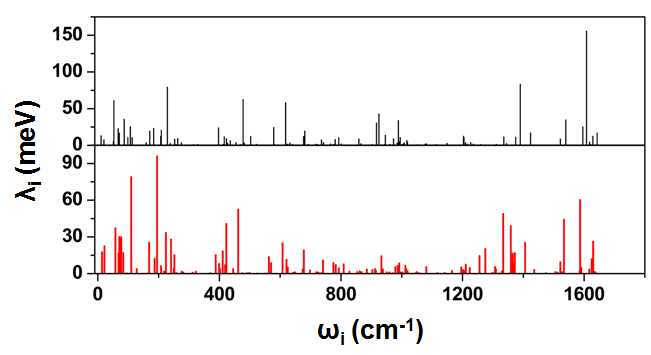


**Figure S1**. (a) Frequency dependence of reorganization energies for neutral and anionic oxided rubrene upon electron-transfer process; the black lines represent neutral oxided rubrene, the red lines represent anionic oxided rubrene.

**Figure S2.** Charge-hopping pathways T1, T2, T3, T4 (T-type), P1, and P2 (P-type) for rubrene and fm-rubrene.

**Figure S3.** HOMOs (0.02 au) for T dimer of rubrenes viewed along short molecular axis direction (a), and along long molecular axis direction (b); LUMOs (0.02 au) for T dimer of rubrenes viewed along short molecular axis direction (c), and along long molecular axis direction (d).

**Figure S4.** HOMOs (0.02 au) for T dimer of fm-rubrenes viewed along short molecular axis direction (a), and along long molecular axis direction (b); LUMOs (0.02 au) for T dimer of fm-rubrenes viewed along short molecular axis direction (c), and along long molecular axis direction (d).

**Figure S5.** Relative contributions of various intermolecular contacts to the Hirshfeld surface area in rubrene, fm-rubrene, and oxided rubrene.

**Figure S6.** Equations used to measure interaction energies of intermolecular F···H/H···F in fm-rubrene crystals.


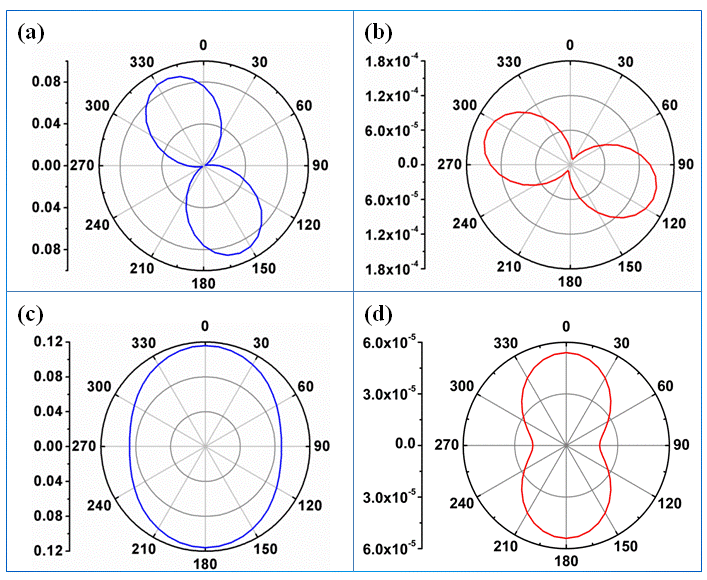


**Figure S7.** Calculated angle-resolved anisotropic hole mobilities (a) and electron mobilities (b) in ac plane; and calculated angle-resolved anisotropic hole mobilities (c) and electron mobilities (d) in bc plance for oxided rubrene.
